# Supplementary material for: Complex‐centric proteome profiling by SEC‐SWATH‐MS
Source: Mol Syst Biol. 2019 Jan 14;15(1):e8438. doi: 10.15252/msb.20188438 (PMC6346213; doi:10.15252/msb.20188438)
Supplement: Supplementary file 8 — Dataset EV7 [file MSB-15-e8438-s008.zip › feature_plots_string/Q9NZQ3.pdf]

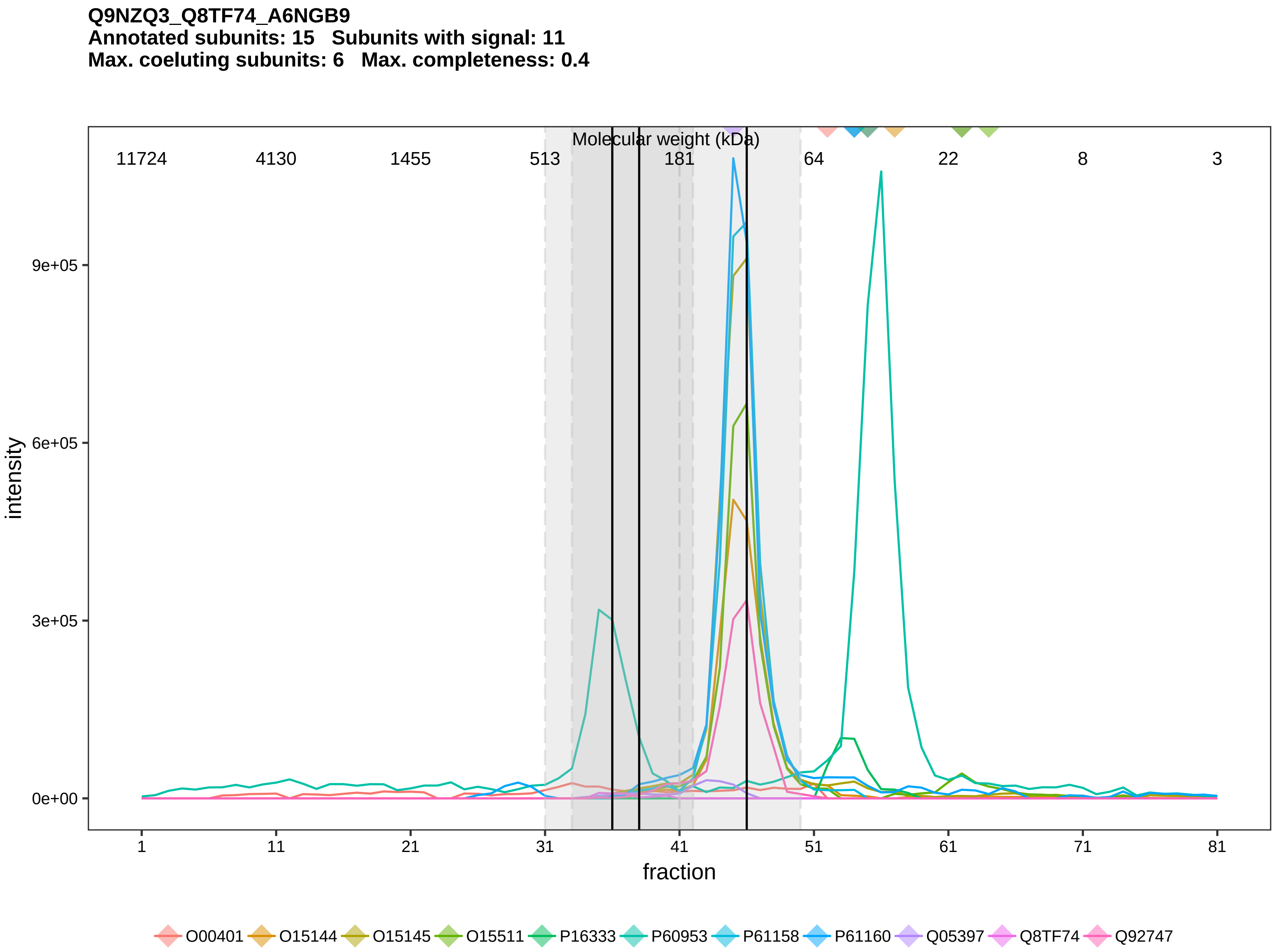
Q9NZQ3_Q8TF74_A6NGB9
Annotated subunits: 15   Subunits with signal: 11
Max. coeluting subunits: 6   Max. completeness: 0.4
Molecular weight (kDa)
11724
4130
1455
513
181
64
22
8
3
9e+05
6e+05
3e+05
0e+00
intensity
1
11
21
31
41
51
61
71
81
fraction
O00401
O15144
O15145
O15511
P16333
P60953
P61158
P61160
Q05397
Q8TF74
Q92747
